# A National Pension Fund for Hospital Nurses and Officials

**Published:** 1887-10-15

**Authors:** Henry C. Burdett

**Affiliations:** Chairman of the Executive Committee of The Hospitals Association, and Founder of The Home Hospitals Association for Paying Patients


### A National Pension Fund for Nurses and Hospital Officials.

By Henry C. Burdett,

Chairman of the Executive Committee of The Hospitals Association, and Founder of The Home Hospitals Association for Paying Patients.

During the last five years there has arisen a feeling, which has gathered strength and volume year by year, in favour of the establishment, upon a financially sound and adequate basis, of a fund out of which all who are engaged in ministering to the requirements of the sick in this country, who are not otherwise provided for, may obtain on provident principles annuities or pensions, and possibly temporary and permanent sick-relief when they themselves are struck down by illness or incapacitated by age from further labour in the cause of suffering humanity. There is indeed reason to believe that the time has arrived when the outlines of a scheme, which will adequately meet the requirements of those referred to, may be usefully formulated and discussed. The soundness of this view is testified to by the widespread interest this proposal has excited amongst all classes of workers, in every grade of hospital and institution, who would be likely to benefit by the establishment of a National Pension Fund for Hospital Officials and Trained Nurses.

**The Existing Provision.**

At the outset it may be well to inquire what provision exists at the present time for pensioning any portion of the great army of workers who labour in the hospital field, using the word hospital in its widest and most extensive sense. First of all, those who devote their time and attention to the care of the insane in public asylums may be provided for by the authorities, Parliament having sanctioned the devotion of public moneys for this purpose. In the same way the Metropolitan Asylums Board *employés* and those who attend to the sick in Poor-law Asylums may be provided for, at the discretion of their employers, from public funds. Then there is the Trained Nurses' Annuity Fund, for which we are indebted to Lady Bloomfield's munificence, which provides a pension of £15 a year to some fifteen women who have been engaged in medical nursing for fifteen years, on their attaining fifty years of age, preference being given to those who have saved £15, which sum each annuitant must pay, on election, into the general fund. Lastly, there are the few hospitals and nursing institutions which individually endeavour to make some provision for their nurses. At Guy's Hospital there is a scheme which was established in 1851, which offers sisters and servants in the employ of the hospital, who contribute to it, one-half the amount that will be necessary according to his or her age, the other half being contributed by the governors as an addition to the wages, or as a reward to those in their service. Every person in the service of the hospital under fifty years of age is required to contribute, but those dismissed for misconduct forfeit all interest in the Fund. The average of superannuation is estimated at sixty-five years. Those who choose can, by paying a higher annual rate, take their money out with 3 per cent. simple interest on leaving, or in the event of death, this money is paid to the friends. Dr. Steele states that very few of the nurses have availed themselves of this Fund, as the majority leave after a short period of training, to take appointments in other hospitals, or to become private nurses. At the Middlesex Hospital all fees for training lady probationers have been placed to a separate fund, from which it is proposed to pay pensions after fifteen years' service. The amount thus received appears to have been so large as to warrant the committee in spending many hundreds of pounds in erecting a Nurses' Home in juxtaposition to the hospital buildings, for the accommodation of the staff engaged in private nursing. The St. John's House Sisterhood,* the Institution of Nursing Sisters, Devonshire Square and the All Saints' Sisterhood make arrangements for old and disabled sisters after fifteen years' service. Such pensions are intended as a provision for old age when the sisters are unable to maintain themselves, and as supplementary to their own savings. Among provincial institutions tentative arrangements for pensioning nurses appear to be in existence at Bath and Cambridge, but particulars are not forthcoming; whilst in several institutions, as, for instance, at the Birmingham General Hospital and the Worcester General Infirmary, pensions have been granted to sisters and nurses after twenty-five years' service, on the ground of long connection with the hospitals. Schemes have been projected, and are under consideration, at the Westminster Hospital, and at the Carlisle Infirmary; whilst at the Exeter Hospital the Pension Fund lapsed because "it proved ruinous to the Institution." A similar attempt was also unsuccessful at Sheffield. In the last two instances the absence of rules and adequate care at the outset no doubt proved fatal, as might have been expected, to the scheme. It will be gathered from the above facts that for all practical purposes no attempt has yet been made to provide adequately for the sustenance and care of nurses, much less of hospital officials, when permanently invalided, or when too old for duty; such provision as exists being very limited in quantity, and not readily available, because where existent removal to a new sphere of duty disqualifies the members. A National Fund would be conceived on sufficiently broad lines to include the greatest number of workers in the hospital field, whilst it would offer them the largest advantages possible, and at the same time afford them adequate guarantees of financial stability.

**What the Scheme should Include.**

It is not necessary at the present stage to elaborate the complete scheme, but it will be sufficient to lay down the general principles on which the National Fund should be established. The details can best be arranged later on, after full consideration, when it is decided what benefits are desired, and can properly be provided. Initially it must be stated that the Fund must be so organised as to be entirely self-supporting, that is, the plan must be such as to ensure permanence and solvency, apart altogether from any donations which may be received from non-members. In other words, the scale of contributions levied on members should be so constructed as in themselves to provide the benefits promised, and to defray working expenses. Although the Fund would thus be independent of outside aid, it must be so conceived as to offer facilities—

1. To the Committees and Managers of Public Institutions to pay at their discretion a portion of the premiums for the whole or any members of the staff they employ.

2. To members of the general public, or to anyone interested in hospital officials or nurses as a class, who desire to give a donation or lump sum to the Fund, which would be applied to provide bonuses to all the members equally.

3. To patients and others who, having received invaluable aid in the day of sickness from a nurse or nurses, could, by giving a donation to the Fund to be applied to her benefit, either (*a*) lessen the amount of her annual contribution, or (*b*) increase the amount of pension she would receive at a given age.

It is necessary to insist upon the adoption of the principle

* Sixty of these nurses have registered their names as wishing to join the National Pension Fund.

that the scale of contributions levied on members must be so constructed as in itself to provide the benefits promised, because, although it necessarily increases the amount which each member who joins the Fund will have to contribute, financial stability must be secured. Besides, a member's contribution could be reduced, or her benefits increased, by the contribution or donation of the employer, or the patient or friend of any individual member who might voluntarily contribute to the Fund as a whole.

Steps so far taken.

Fifteen years' continuous residence in various hospitals and an active participation in their management having convinced the writer of the necessity for a National Pension Fund, and the soundness of the principles above enunciated, he proposed in the autumn of 1883 to collect information and data for the actuary's guidance in drawing a scheme. This work has been continuously pursued from that date to the present time, the whole of the four years being devoted to the attempt to educate public opinion and to organise all that was valuable in the feeling known to exist amongst hospital officials and nurses who would be eligible to participate in such a Fund. The recent announcements that Her Majesty the Queen had decided to devote the Women's Jubilee Offering, amounting to £75,000, to the benefit of nurses and nursing institutions, has naturally given such an impetus to this movement that the principles of the proposed scheme may at length be published with advantage. This decision of Her Majesty may have a material influence on the constitution of the Fund, because a contribution of £20,000 would enable an Annuity Fund to be established according to law, which could grant annuities to any amount, and so include the officials of all ranks in the scheme. Otherwise the Fund would have to be registered under the Friendly Societies' Act, and no annuity could exceed £50 per annum—unless, of course, £20,000 was subscribed by the general public. The labour and expense would be considerable, and much delay must necessarily ensue in the latter case. Then again, if the Queen should decide to support the Pension Fund scheme, it might be called Queen Victoria's Bounty, and thus perpetuate her Jubilee for all time whilst conferring inestimable benefits upon probably the most deserving class of workers in the country.

The Numbers who would be likely to Participate.

At the present time there are in England some five hundred general and special hospitals, three hundred cottage hospitals, and probably as many nursing institutions, besides those located in Scotland and Ireland. During the last twenty years a system of nursing has been developed which has led to young women of twenty years of age and upwards being taken as probationers into the children's hospitals at wages of from £12 to £18 a year. At the general hospitals the age of probationers is usually increased to 25, and not infrequently a probationer from the children's hospital is drafted to a larger hospital, where the scale of wages is increased from £20 to £25 per annum. Many of the better and more capable of these women are in process of time promoted to be sisters or head-nurses, with salaries of from £30 to £50 per annum. Sometimes sisters are again promoted to be lady-superintendents or matrons, at incomes of from £50 to £120 per annum. To become a trained nurse a woman must necessarily attach herself in the first instance to a hospital where she can be trained and receive a certificate, and consequently the nursing staff of every properly constituted nursing institution which supplies private families, as well as that of the special and cottage hospitals, are drafted mainly from the larger hospitals from time to time.

It is difficult to estimate, but from all I can learn, I think I am within the mark in stating that there are some 15,000 nurses in this country to-day, of whom from one-third to one-half are certificated or hospital-trained nurses. During the last four years some 2,500 hospital officials and nurses have intimated their desire to join a National Pension Fund. With the view of testing this feeling thoroughly, The Hospitals Association recently opened a register at Norfolk House, Norfolk Street, Strand, W.C., where, during the last few weeks, in consequence of this fact being announced in the columns of THE HOSPITAL, upwards of 1,000 names have been registered. I think, therefore, we may assume that evidence is forthcoming to prove that a very considerable number of persons engaged in attending the sick are anxious to devote their lives to the work, and, as cautious and reasonable people, to secure an adequate provision for themselves when disabled by age or other infirmity.

What is to be Provided.

In the course of these inquiries a mass of information has been elicited as to what provision is desired by the nurses from the institution of a Pension Fund. First of all, for reasons which will be stated later on, it seems to be essential that no nurse shall be eligible to join the Pension Fund who has not received adequate training. That is to say, whether the applicant is a hospital nurse, a private nurse, or a midwife, to entitle her to membership she must produce evidence to the satisfaction of the managers of the Fund that she is a trained, *i.e.*, a certificated nurse. In the same way hospital officials who desire to avail themselves of the Fund will also have to produce evidence that their experience and position entitle them to join as members. Every member will be entitled to benefits in proportion to the amount which he or she contributes to the Fund. For example, it is proposed that a hospital secretary may elect to enter for a pension of £100 per annum, or any sum up to £300 per annum, a matron for any sum up to £250 per annum, and any other official for any sum up to £100 per annum, on attaining a certain age. All that will be required is that the annual premiums shall be paid with regularity as they fall due year by year; or that a single contribution or a lump sum shall be paid into the Fund, in accordance with the tables prepared by the actuary. This is the only distinction that can be drawn between any of the members who desire to participate. All, with possibly the exception of midwives, whose case is a special one, would pay at the same rate, though the amount of the contributions of each individual would depend upon the benefits which they desired to ultimately derive from the Fund. Another point which has given rise to much discussion is the establishment of a Sick Fund. The general result seems to prove that those nurses who are engaged in hospitals and similar institutions, where they are provided for when temporarily ill, will not require the assistance of such a fund except for permanent disablements; although nurses attached to nursing institutions would find temporary as well as permanent sick-pay essential to their well-being and comfort.

The scheme will therefore include sick-relief for twelve months' continuous illness; or, if members prefer it, they can, by paying an increased rate, secure a fixed weekly payment up to fifty years of age, when permanently invalided. Each member could enter for sick-relief or not, at discretion, on making the necessary payments to the Fund, and passing such a medical examination as may be thought necessary by those responsible for the management. Many questions have been asked as to the extreme limit of age on joining. This is again a matter which can only be adjusted on financial principles. Any nurse may join up to forty years of age on paying the necessary premium, or she can compound that premium by handing over her savings to the Fund as a lump payment, fixed at an amount proportionate to the annuity or pension she desires ultimately to receive. It is not proposed to give a pension to any nurse before she attains the age of fifty years, and those who have good constitutions may at their option enter at a reduced premium by postponing the date when they will be entitled to a pension, or by waiting till they attain 55 or 60 years, the latter being the Government period.

*(To be continued.)*

